# A clinical observation study on the effect of needle-free insulin syringe on blood glucose control and well-being index in patients with early-onset type 2 diabetes mellitus

**DOI:** 10.3389/fendo.2023.1137179

**Published:** 2023-02-14

**Authors:** Xiaolong Jin, Qiuying Sun, Chenying Yue, Junming Han, Xinli Zhou, Qingbo Guan, Xu Zhang

**Affiliations:** ^1^ Department of Endocrinology, Shandong Provincial Hospital Affiliated to Shandong First Medical University, Jinan, Shandong, China; ^2^ Shandong Clinical Research Center of Diabetes and Metabolic Diseases, Shandong Provincial Hospital Affiliated to Shandong First Medical University, Jinan, Shandong, China; ^3^ Shandong Key Laboratory of Endocrinology and Lipid Metabolism, Shandong Provincial Hospital Affiliated to Shandong First Medical University, Jinan, Shandong, China; ^4^ Shandong Prevention and Control Engineering Laboratory of Endocrine and Metabolic Diseases, Shandong Provincial Hospital Affiliated to Shandong First Medical University, Jinan, Shandong, China

**Keywords:** Type 2 diabetes mellitus, early-onset, needle-free syringe, novo pen, transient scanning glucose monitoring system, well-being index

## Abstract

**Objective:**

To explore the effect of using needle-free insulin syringe on blood sugar control and well-being index in patients with early-onset type 2 diabetes mellitus.

**Methods:**

A total of 42 patients with early-onset type 2 diabetes mellitus treated with insulin aspart 30 injection in a stable condition in the Endocrinology Department of a tertiary hospital from January 2020 to July 2021 were randomly divided into two groups, one group received insulin pen injections followed by needle-free injections, and the other group received needle-free injections followed by insulin pen injections. Transient scanning glucose monitoring was performed during the last two weeks of each injection modality phase. Comparison of the two injection methods in terms of test indicators and differences in injection site pain scores, the number of red spots on the skin at the injection site and the number of bleeding spots on the skin at the injection site.

**Results:**

The FBG of the needle-free injection group was lower than that of the Novo Pen group (p<0.05); the 2-hour postprandial blood glucose of the needle-free injection group was lower than that of the Novo Pen group, but there was no statistical significant difference. The amount of Insulin in the needle-free injector group was lower than that in the Novo pen group, but there was no statistical significant difference between the two groups. The WHO-5 score of the needle-free injector group was higher than that of the Novo Pen group(p<0.05); the pain score at the injection site was lower than that of the Novo Pen group (p<0.05). The number of skin red spots using the needle-free syringe was more than that of the Novo pen group(p<0.05); the number of skin bleeding at the site of injection was similar between the two injection methods.

**Conclusion:**

Compared to traditional insulin pens, subcutaneous injection of premixed insulin using a needle-free syringe is effective in controlling fasting blood glucose in patients with early onset type 2 diabetes and is less painful at the injection site. In addition, blood glucose monitoring should be strengthened and insulin dosage should be adjusted in a timely manner.

## 1 Introduction

Diabetes has become a global and serious social health problem. At present, there are about 425 million adults with diabetes worldwide, and it is estimated that by 2045, this number will grow to 629 million (1). The increased prevalence of early-onset type 2 diabetes is particularly pronounced in the rapidly growing diabetes population (2). Early-onset type 2 diabetes refers to type 2 diabetes diagnosed at age ≤ 40 years old (3). According to the data of the Global Diabetes Map (8th Edition) released by the International Diabetes Federation (IDF) in 2017, the top three countries and regions with the highest prevalence of early-onset type 2 diabetes are the Middle East and North Africa (4.1%), the United States. (4.0%) and China (3.6%). According to the latest epidemiological research results in China, the prevalence rate of early-onset type 2 diabetes is about 10% to 15% among diabetic patients who have been treated. Further studies (4, 5) have found that early-onset type 2 diabetes has more severe metabolic disorders, a higher risk of diabetic macrovascular disease and microvascular disease and a greater impact on life expectancy. Hence, critical attention must be given to patients with early-onset type 2 diabetes in order to strictly control blood sugar and reduce the occurrence of diabetic complications.

Patients with early-onset type 2 diabetes are more likely to use insulin to lower blood sugar, regardless of initial intensive insulin therapy or as the disease progresses, with the decline of pancreatic β-cell function. However, in the course of insulin therapy, problems such as pain, discomfort, fear, and adverse reactions caused by traditional needle injections will affect the acceptance and compliance of insulin therapy, and adversely affect the blood sugar control and quality of life of patients (6). “Guidelines for Diabetes Drug Injection Technology in China (2016 Edition)” recommends the use of needle-free syringes for insulin therapy in diabetic patients (7). Thus, the development of insulin injection devices prompts clinicians to re-examine the choice of hypoglycemic treatment strategies and injection devices.

Various researchers have conducted related studies to explore the differences in pharmacokinetics and clinical efficacy between needle-free injection of insulin and traditional subcutaneous injection (8, 9). For example, the “FREE study” conducted by Ji Linong in ten (10) research centers in China showed that, compared with needle-based insulin injection, needle-free insulin injection can better reduce a patients’ glycosylated hemoglobin, lower the dosage of insulin required to achieve similar effect, less pain for patients, significantly lower incidence of adverse reactions such as induration at the injection site and higher patient contentment. At present, there is no clinical research on needle-free injection for early-onset type 2 diabetes. In-depth research on it will explore more effective insulin treatment methods and provide a better therapeutic approach in the treatment of early-onset type 2 diabetes.

The instantaneous scanning glucose monitoring system (flash glucose monitoring, FGM), uses advanced sensor-based technology whereby glucose data can be obtained through scanning. It is a tool for continuously recording 14-day blood glucose values ​​and predicting the trend of blood glucose fluctuations, which can be used to evaluate and adopt treatment strategies to reduce blood glucose fluctuations (11). Based on the hospitals version of FGM data, this study took patients with early-onset T2DM as the research subjects, and compared the effect of needle-free injector and Novo pen subcutaneous insulin injection on blood sugar control and well-being index in patients with early-onset type 2 diabetes through a randomized cross-over study before and after self-control design. This was done in order to provide further clinical evidence in the treatment of early-onset type 2 diabetes using needle-free injection.

## 2 Subjects and methods

### 2.1 Subjects selection

The research subjects were selected between January 2020 to July 2021 in the the endocrinology department of a tertiary hospital in Shandong Province and were randomly divided into two groups. All the subjects were confirmed to be Type 2 diabetes patients.

### 2.2 Diagnostic criteria

#### 2.2.1 Inclusion criteria

The following requirements had to be met for any subject to be included in the study:

1. Meet the 1999 WHO diabetes diagnostic criteria;
2. Aged between 18 and 40 years old;
3. using or be willing to use premixed insulin therapy;
4. Body Mass Index of 18Kg/m^2^≦BMI≦32Kg/m^2^;
5. A hemoglobin A1c (HbA1c) in the range 7.5% - 11%;
6. Have read and signed the informed consent form.

#### 2.2.2 Exclusion criteria

The following requirements had to be met for any subject to be rendered unfit or disqualified for the study:

1. Confusion or language barrier;
2. Patients with heart, lung, liver, kidney and other organ failure, acute cardiovascular and cerebrovascular diseases or severe sequelae;
3. Type 1 diabetes patients;
4. Individuals with psychiatric history;
5. People with combined acute and chronic severe diabetes complications;
6. Any patient with a history of insulin allergy;
7. Individuals with secondary diabetic complications such as acute pancreatitis, abnormal thyroid function, etc.;
8. Pregnancy and lactation mothers;
9. Cancer patients or people with history of autoimmune diseases;
10. Patients with irregular diet.

A total of 50 patients with type 2 diabetes mellitus and on premixed insulin regimens initially joined the study, among which two (2) patients fell off the flash glucose monitoring sensor, four (4) patients sought other treatment options midway, and two (2) patients did not complete the entire research process and dropped out. A total of forty-two (42) patients completed the study. The general information and metabolic status of the subjects before the intervention are shown in **Table 1**.

**Table 1:** General data and metabolic indexes of subjects before intervention (n=42).

Subject Parameters	Data
Male (number)	38
Female (number)	4
Age (years)	34.95 ± 4.62
Course of disease (years)	1.64 ± 1.71
BMI (Kg/m^2^)	27.63 ± 3.67
Fasting blood glucose (mmol/L)	5.77 ± 1.41
2h postprandial blood glucose (mmol/L)	7.91 ± 1.57
HbA1c (%)	8.44 ± 3.51
TG (mmol/l)	2.84 ± 1.77
TC (mmol/l)	6.29 ± 2.11
HDL-C (mmol/l)	1.06 ± 1.34
LDL-C (mmol/l)	4.12 ± 2.15
SBP (mmHg)	127.21 ± 12.36
DBP (mmHg)	79.64 ± 9.72

### 2.3 Methods

#### 2.3.1 Study design

**Figure 1:**
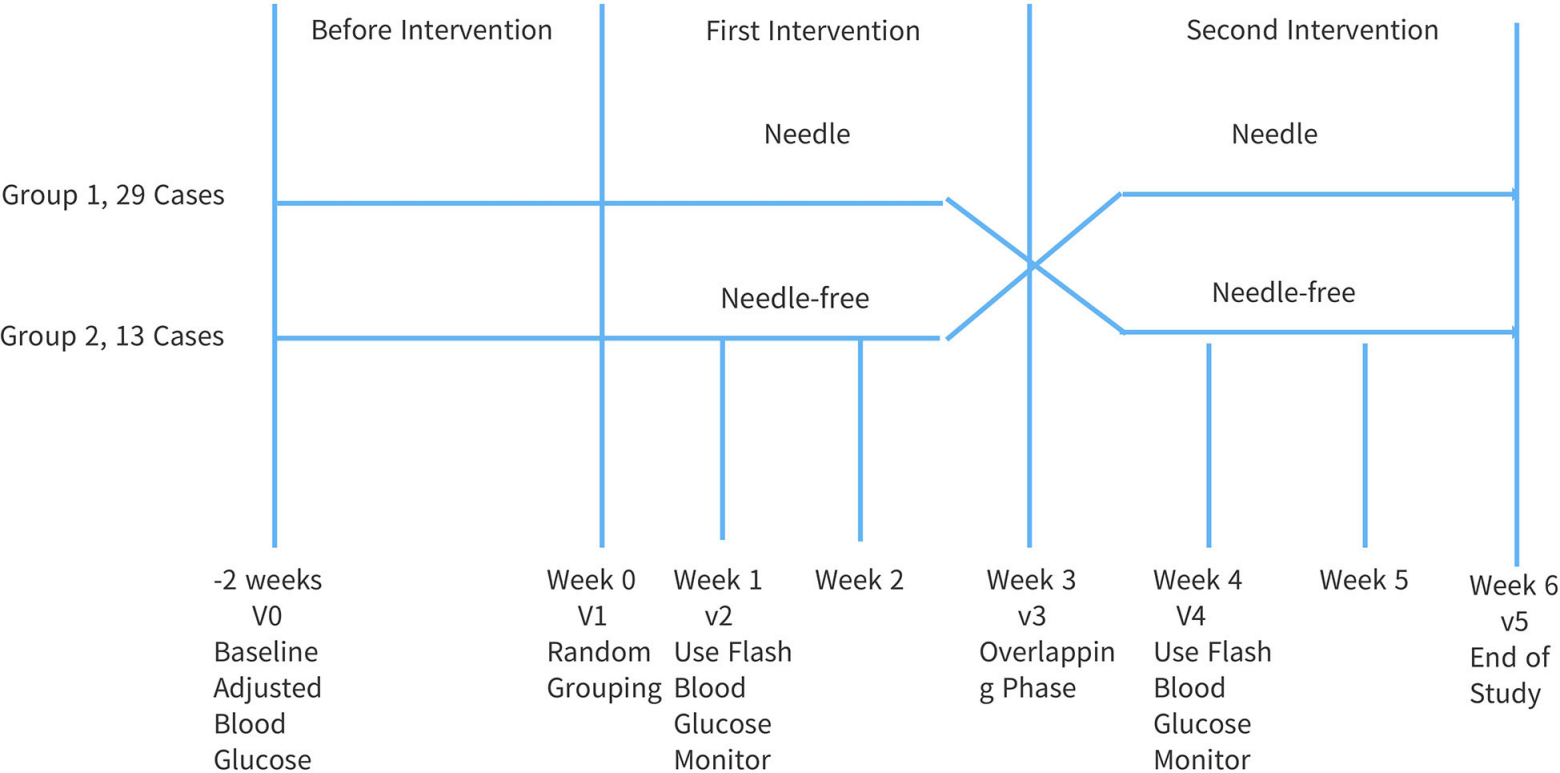
Flow chart of cross test.

This study adopted a prospective, randomized, open, cross-controlled design, see **Figure 1**. The overall intervention time was 8 weeks. Forty-two (42) patients were randomly divided into two groups with one group receiving insulin pen injections followed by needle-free injections, and the other group receiving needle-free injections followed by insulin pen injections. Before the intervention, the subjects were treated in the early stage, blood sugar was monitored and medications were adjusted for 2 weeks. This was done to ensure that the research was non-bias. Intervention Phase 1: After being discharged from the hospital, the two groups of subjects were given injections without needles and injections with needles, respectively, for three (3) weeks. Intervention Phase 2: three (3) weeks after being discharged from the hospital, the injection methods of the two groups were reversed for a further duration of three (3) weeks.

Among them, the needle-free injection used was a Beijing fast-comfort (QS-M) needle-free syringe; the syringe injection used was a Novo Nordisk Novo Pen ⑤ and a disposable insulin syringe of size 31G 5mm×0.25mm from Becton, Dickinson (BD) and company. Intervention Phase 1 and Intervention Phase 2 patients in both groups were treated with insulin aspart 30 (pen core/refill) manufactured by Novo Nordisk, Denmark. The lower abdomen was selected as the route of administration for the two different ways of insulin injection. Peripheral blood glucose was monitored with an Essence blood glucose meter, transient scanning glucose monitoring was performed two (2) weeks after the first and second intervention phases. Health records and management was established for all the patients and strengthened. During the study period, the same health guidance was given to all the subjects, including insulin injections, diet control, regular follow-up, exercise and outpatient review. This was done to ensure effective data gathering.

#### 2.3.2 Comparison of various parameters

We did an evaluation and comparison of the effects of the two injection methods on fasting blood glucose (FBG), postprandial 2h blood glucose (2hPPG), time in range (TIR), World Health Organization-Five Well-Being Index (WHO-5), Insulin dosage [U/(kg·d)], pain score at the injection site, number of skin red spots at the injection site, number of skin bleeding at the injection site, times of hypoglycemia and time below range (TBR).

### 2.4 Statistical analysis

Using SPSS20.0 statistical software, the measurement data conforming to the normal distribution are expressed as ( $\bar{x}\pm sd$ ). All comparisons between the two groups were first analyzed using a one-sample t test and did not conform to the normal distribution. As such, comparison between the two groups was performed by the non-parametric test where p<0.05 was considered statistically significant.

## 3 Results

### 3.1 Comparison of the effects of the two injection methods on the dosage of FBG, 2hPPG, TIR, TBR and insulin

The patients in the two groups received subcutaneous insulin injection with Novo pen and needle-free syringe respectively. The fasting blood glucose (FBG) of the needle-free syringe was lower than that of the Novo pen group, and the difference was statistically significant (p<0.05); the 2h postprandial blood glucose (2hPPG) was lower in the needle-free group than that of the Novo pen group, but there was no statistically significant difference. In addition, no statistically significant difference was observed between the two groups for the TIR and TBR group. The amount of Insulin in the needle-free injector group was lower than that in the Novo pen group, but the difference was not statistically significant. A detailed account of the results is presented in **Table 2**.

**Table 2:** Comparison of the effects of two the injection methods on blood glucose related indexes ( $\bar{x}\pm sd$ ).

Group	Subjects (n)	FBG (mmol/L)	2h PPG (mmol/L)	TIR (%)	TBR (%)	Insulin Dosage [U/(kg·d)]
Needle-Injection Group	42	6.10 ± 1.74	7.94 ± 1.94	0.66 ± 0.19	0.13 ± 0.18	0.28 ± 0.08
Needle-free Injection Group	42	5.64 ± 1.59	7.67 ± 2.58	0.66 ± 0.17	0.12 ± 0.16	0.27 ± 0.28
*T values*		-2.338	0.887	-0.086	0.616	1.255
*P values*		0.024	0.380	0.932	0.542	0.217

### 3.2 Comparison of the effects of the two injection methods on the WHO-5, the pain score at the injection site, the number of red spots on the skin at the injection site and the number of bleedings at the injection site.

The WHO-5 score of patients using needle-free syringes was averagely higher than that of the Novo Pen group with a statistically significant difference (p<0.05). The pain score at the injection site was lower in the needle-free syringe group than that of the Novo Pen group, and the difference was statistically significant (p<0.05). The number of skin red spots using the needle-free syringe was more than that of the Novo pen group with a statistically significant difference (p<0.05). The number of skin bleeding was similar between the needle-free syringe group and the Novo Pen group. The data is presented in **Table 3**.

**Table 3:** Comparison of the effects of the two injection methods on satisfaction indicators ( $\bar{x}\pm sd$ ).

Group	Subjects (n)	WHO-5 score	Pain score	Skin red spots (times)		Skin bleeding (times)
Needle-Injection Group	42	73.67 ± 16.46	2.57 ± 1.93	0.19 ± 0.67		0.29 ± 0.64
Needle-free injection group	42	81.38 ± 15.70	1.83 ± 1.41	0.79 ± 1.22		0.45 ± 1.04
*T values*		4.387	4.109	2.909		2.011
*P values*		0.000	0.000	0.006		0.051

## 4 Discussion

Needle-free injectors use the principle of high-pressure jets, and mainly adopt transdermal dispersion drug delivery technology. Without the aid of needles, the liquid drug is ejected out from a small aperture at high speed (flow rate is generally greater than 100m/s) to reach the subcutaneous tissue with insulin distribution averagely higher than when a needle injection is used. This provides a very conducive area for maximal and complete absorption of insulin under the skin into bloodstream (12). In this study, a transient scanning glucose monitoring system was used to evaluate the effect of needle-free injection and needle injection within a 24-hour blood glucose fluctuations period in patients with early-onset type 2 diabetes mellitus. A very dynamic blood glucose data is attainable using the flash blood glucose (FBG) monitoring system, and the data obtained is beneficial in providing a more accurate trend and regularity of the changes in glucose occurring in a patient’s body.

As a group that cannot be ignored, patients with early-onset type 2 diabetes often neglect the control of diabetes because they are busy with work and have the impression that they are in good health, thus causing an aggravation in the decrease of their β-cell function (13). Biphasic insulin aspart 30 is composed of 30% insulin aspart and 70% protamine insulin aspart, which can better meet the needs of patients for mealtime and basal insulin. The use of needle-free syringes to inject short-acting and rapid-acting insulin therapy can significantly shorten the time of onset of insulin, reverse the glycotoxic effect of hyperglycemia on β cells and significantly enhance the hypoglycemic effect (14). In the research conducted, the FBG of patients the patients using needle-free injectors was significantly lower than that of the Novo Pen group (p<0.05).

Time in range (TIR) refers to the time or percentage of glucose in the target range within 24 hours. It is a new indicator of blood glucose control that has attracted much attention in the field of diabetes. TIR can more intuitively and visually reflect the blood glucose fluctuation status of patients throughout the day (15). The WHO-5 scale was developed by the Danish scholar Bech.P and revised by the WHO Collaborative Center for Psychological Research (16, 17). It is the most widely used clinical scale to evaluate the well-being of diabetic patients. The scale can reflect the individual’s mood or sense of well-being in the past two (2) weeks. It includes 5 items, each of which is divided into 6 levels, with a total score of 0-100 points. The higher the score, the stronger the sense of well-being (18). The score is graded as follows: ≤28 points for possible depression or low quality of life, 29-50 points for diminished sense of well-being and >50 points for happiness or contentment (19).

The WHO-5 scale was scored before the intervention, before the switch and at the end of the study. It must be noted that all the questions were willingly answered and filled in by the patients. The Numeric Rating Scale (NRS) is the most widely used unidimensional assessment scale (20). The system is based on a principle where a straight line is divided into ten (10) parts on average, and numbers from 0 to 10 is used at each point to determine the degree and aggravation of pain. The scaling is as follows: 0 indicates no pain; 1-3 indicates mild pain; 4-6 for moderate pain and 7-10 for severe pain. The patient is asked to circle the number that best represents their own level of pain. This evaluation form is more commonly used internationally. During the study, patients scored and recorded their own NRS scores after daily insulin injections. The number of skin red spots, skin bleeding and times of hypoglycemia were recorded by the patients and the data obtained is seen as fair as a consent form was signed.

A significant difference was observed in a meta-analysis (21) conducted on a subgroup of patients with diabetes mellitus who used insulin aspart 30. The fasting blood glucose (FBG) in the needle-free injector group was significantly lowered (MD = -1.31, 95%CI (-1.83, -0.78), p<0.00001) in comparison with the group that used the Novo pen. The results obtained is consistent with what we had in our report. In this study, there was no significant difference between the 2h postprandial blood glucose and TIR between the two groups, which may be attributed to the small sample size and short observation time.

The transdermal diffuse flow technology of needle-free injectors can increase the bioavailability of insulin (22) and help reduce the insulin dosage required to achieve similar therapeutic effect. As such, when changing to needle-free injectors, the insulin dose should be adjusted in time to avoid increasing the risk of hypoglycemia in patients. In this study, there is no statistically significant difference between the two groups of in terms of time below range (TBR), which is consistent with the previous research results that there is no difference in the incidence of hypoglycemia between needle-free injection and needle injection (23). In addition, it also shows the predictive effect of the scanning glucose monitoring system on the patient’s glucose trend (24). In research conducted by Xie et al., the study found that when patients with type 2 diabetes change their insulin pens to needle-free injectors, regardless of the dosage form (fast-acting, short-acting, premixed) insulin, when the original insulin dose is maintained, the blood glucose levels of the patients dropped sharply within 0.5h, and hypoglycemia occurred frequently. In this regard, clinical therapists should strengthen the management of out-of-hospital diabetic patients who use needle-free injection for the first time, and conduct a comprehensive assessment based on the patient’s blood sugar status and the body’s absorption of insulin to avoid the occurrence of hypoglycemia. In this study, the amount of insulin in the needle-free injector group was lower than that in the Novo pen group, but the difference was not statistically significant.

Needle-free injection is not painless and non-invasive, but minimally painful and minimally invasive. When the medical composition in the insulin injection penetrates the human epidermis, a small injection mark will be left, but since only the medicinal liquid enters the skin, and the injection depth is approximately 4-6mm with few nerve endings in this range, no obvious irritation will occur (25). Nonetheless, due to differences in individual physique, patients feel slightly different when injected. In this study, patients who used needle-free syringes had less pain at the injection site than needle-based injections, and the difference was statistically significant (p<0.05). The number of skin bleeding was similar between the two injections, but the number of red spots on the skin was more pronounce with the needle-free syringe than with the Novo pen, and the difference was statistically significant (p<0.05). The patients in this study had fewer daily injections, and the red spots or bleeding at the injection site disappeared after three (3) to five (5) days. There was a higher level of patient compliance and better injection experience among all the subjects by the end of the study.

Needle-free injection does not use needles, which not only reduces the patient’s physical pain, but also eliminates the patient’s psychological fear of injection needles. The WHO-5 scale can reflect an individual’s mood or sense of well-being. In this study, the WHO-5 score of the needle-free injection group was higher than that of needle-based injection, and the difference was statistically significant (p<0.05). Patients were more willing to accept needle-free injection, so the well-being index was significantly improved. Studies have shown that the application of needle-free syringes can improve patients’ depression as well as their quality of life (26). Although this study proved a point in helping determining which of the two injection methods is more ideal, it had certain limitations. The sample size of the study was mall and the observation time not long enough. For this reason, this study employed random grouping and cross-control design to reduce the difference between groups and in addition, enhance the feasibility of comparison between the two groups. In the future, the sample size will be increased, the observation time will be extended and further in-depth research will be conducted in order to gain a more comprehensive understanding of the advantages of needle-free injection in the administration of insulin.

In conclusion, compared with traditional insulin pens, the use of needle-free syringes and enhanced management can effectively reduce the fasting blood glucose of patients with early-onset type 2 diabetes mellitus treated with premixed insulin. To improve adherence to insulin therapy, critical attention should be paid to adjusting the insulin dose in time to aid in reducing the occurrence of hypoglycemia. Needle-free injection is more suitable for young patients and people who are Trypanophobia and it is particularly important to strengthen injection guidance among patients with early-onset type 2 diabetes mellitus.

## Data availability statement

The original contributions presented in the study are included in the article/supplementary material. Further inquiries can be directed to the corresponding authors.

## Ethics statement

The studies involving human participants were reviewed and approved by Ethics Committee of Shandong Provincial Hospital Affiliated to Shandong First Medical University. The patients/participants provided their written informed consent to participate in this study.

## Author contributions

Conceptualization, methodology, software, validation, formal analysis, writing—original draft preparation, XJ and QS; investigation, resources, data curation, visualization, CY, JH, and XLZ; supervision, project administration, and writing—review and editing, QG and XZ. All authors contributed to the article and approved the submitted version.
